# Outcomes of the Electrothermal Bipolar Vessel Sealing Device and Electrocautery in Modified Radical Mastectomy Patients

**DOI:** 10.7759/cureus.62371

**Published:** 2024-06-14

**Authors:** Ravindran Chirukandath, Sancia R Fernandez, Sharath K Krishnan, Sumin V Sulaiman, Daniel Suting, Soorya Gayathry P., Sreeparvathy R Nair

**Affiliations:** 1 Department of General Surgery, Government Medical College, Thrissur, Thrissur, IND

**Keywords:** vessel sealing devices, bipolar diathermy, ebvs, electrosurgery, mastectomy, breast

## Abstract

Introduction

With the rising trends in breast cancer throughout the world, the traditional modus of intraoperative tissue dissection using a scalpel, scissors, or electrocautery needs to be re-evaluated in the wake of newer modalities, such as electrothermal bipolar vessel sealing (EBVS) devices, which may theoretically reduce the postoperative complications and morbidity in these patients.

Aim and objective

The objective of this study is to compare an EBVS device to electrocautery (diathermy) in modified radical mastectomy (MRM), based on selected intraoperative and postoperative outcomes.

Study procedure

This was a comparative cross-sectional section study that included 60 patients with operable breast cancer (stages I and II, TNM classification, and post-neoadjuvant stage III disease). Patients were divided into two groups for surgery: one group underwent an MRM using the EBVS device (Group A), while the other group had the procedure performed using conventional electrocautery (Group B), as per the surgeon’s choice depending on theatre slot and equipment availability. Intraoperatively, the total operative time, time for raising the flaps, time taken for breast tissue dissection, time for axillary dissection, and blood loss were recorded. Postoperative parameters included total drainage volume, number of days of drainage, seroma formation, and other complications. Patients were followed up for one month after surgery, with early postoperative complications such as wound infection, upper limb lymphedema, seroma, flap necrosis, and nerve injuries being documented.

Results

The groups were found to be comparable in terms of the age distribution, TNM staging, stage grouping, and nodal status of the patients. The EBVS device group demonstrated statistically significant advantages in total operative time, axillary dissection time, flap raising time, breast tissue dissection time, intraoperative blood loss, total drainage volume, and days of drainage. However, no statistically significant difference was found between the two devices in terms of seroma formation, early postoperative complications, and duration of postoperative stay at the hospital.

Conclusion

While the use of EBVS in MRM provides a considerable decrement in the total operative duration, duration of the various steps of surgery, intraoperative blood loss, and postoperative volume and duration of drainage, these devices do not offer an evident advantage in terms of the postoperative complications or morbidity.

## Introduction

Breast carcinoma is the most common cancer amongst women worldwide, accounting for 22% of all cases of malignancies in women, and is the single leading cause of cancer deaths in women worldwide [1]. In India, breast cancer is the most dominant cancer amongst women, with age-adjusted incidence rates as high as 25.8 per 100,000 women and a mortality of 12.7 per 100,000 women [2]. In Indian women, it has shown a steadily rising trend over the years [3]. It becomes of paramount importance to find better treatment modalities, as well as reduce complications associated with these treatment methods.

Surgical resection remains the keystone of breast cancer therapy, even though the specific approach for each case depends on the stage and type of tumor, and includes a multidisciplinary tactic involving surgery, radiation, and chemotherapy. It is crucial to prevent common surgical complications to avoid unnecessary delays in adjuvant treatment and to offer a better recovery period to these ailing patients.

Modified radical mastectomy (MRM) is considered the most common operative procedure performed for advanced breast cancer [4]. Early complications in breast cancer surgery have always been attributed to excessive postoperative drainage, prolonged hospital stay, seroma formation, blood loss, hematoma formation, skin necrosis, and nerve injury [5].

Traditionally, raising the skin flaps, dissecting the breast from the pectoral wall, and performing axillary lymph node dissection have been accomplished using either a scalpel (sharp dissection) or electrocautery. Recently developed methods include the use of UltraCision dissection (harmonic scalpel) and tissue response generation using electrothermal bipolar vessel sealing (EBVS) devices in raising these flaps.

This study was done to evaluate if the theoretical benefits and drawbacks of using EBVS devices in MRM are reflected in the outcomes experienced by patients in a practical setting.

## Materials and methods

This prospective observational study, aimed at evaluating the differences in outcomes of using electrocautery against EBVS devices, was conducted between September 2019 and September 2020 in a single unit in the Department of General Surgery and Surgical Oncology in the tertiary care setup of Government Medical College, Thrissur, India, with a total of 60 participants with operable breast cancer.

After taking a detailed history, conducting a complete physical examination, and performing relevant investigations, consent was obtained from all patients for both the MRM and their participation in the study. All patients underwent mammography or ultrasound of both breasts, along with a metastatic workup to rule out metastasis. Preoperatively, each patient also had a fine needle aspiration cytology (FNAC) or Tru-Cut needle biopsy performed. They were separated into two equal groups, either to undergo MRM using the EBVS device (Group A) or to undergo it using conventional electrocautery (Group B), as per the surgeon’s choice, depending on theater slot and equipment availability.

The total operative time, time for raising the flaps, time for dissecting the breast tissue, time for axillary dissection, and blood loss were measured intraoperatively. All major intraoperative events were meticulously recorded. Postoperative parameters included total drainage volume, days of drainage, seroma formation, and other complications.

## Results

The average age of the subjects in the study population is 52.6 ± 9.4 years with a range between 34 and 70 years. The mean age in the electrocautery group was 52.5 ± 9.081 years and 52.7 ± 9.396 years in the EBVS group.

The distribution of patients with node positivity in each device group was found to be comparable amongst the study subjects (p = 0.302). This is in line with other similar studies that have emphasized keeping nodal positivity rates similar amongst both case and control groups.

The average total intraoperative time for the study population was found to be 62.7 ± 8.579 minutes. Of those who underwent the procedure using electrocautery, the average total intraoperative time was found to be 70.2 ± 3.899 minutes, whereas those patients who underwent the procedure using the EBVS device had their procedures completed in 55.2 ± 4.262 minutes. The result was found to be statistically significant (t = 14.223; p < 0.001).

**Figure 1:**
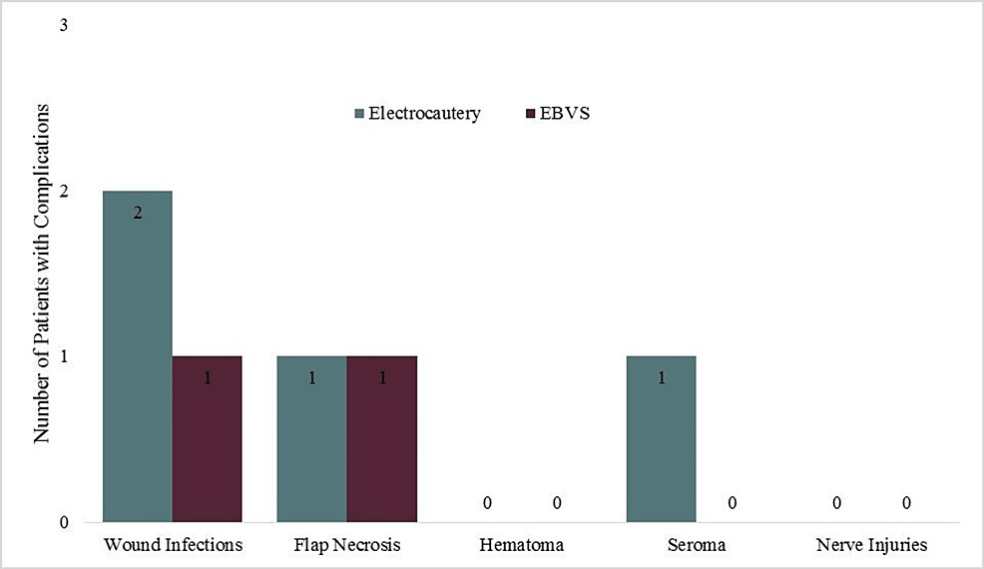
Comparison of average operative time for various steps of MRM MRM: modified radical mastectomy; EBVS: electrothermal bipolar vessel sealing

The average time taken for raising flaps was found to be 24.2 ± 3.729 minutes for the study population. For those who underwent MRM using electrocautery, the average time taken for raising the flaps was found to be 27.37 ± 2.008 minutes, whereas those patients who underwent MRM using the EBVS device had their flaps raised in 20.97 ± 1.752 minutes. This finding proved to be statistically significant (t = 13.155; p < 0.001) (Figure 1).

The key area of concentration was axillary dissection among the study population. The average time taken for axillary dissection was found to be 25.82 ± 3.039 minutes. The duration was found to be 28.33 ± 1.470 minutes for those who underwent the procedure using electrocautery. In contrast, for those who underwent MRM using the EBVS device, the average time taken for axillary dissection was 23.3 ± 1.878 minutes. This was determined to be statistically significant (t = 11.559; p < 0.001) (Table 1).

**Table 1:** Intraoperative timings at various strategic steps EBVS: electrothermal bipolar vessel sealing

Device	Mean total operative time in minutes (SD)	Mean time for raising flaps in minutes (SD)	Mean time for axillary dissection in minutes (SD)	p-value
Electrocautery	70.2 (3.899)	27.37 (2.008)	28.33 (1.470)	<0.001
EBVS device	55.2 (4.262)	20.97 (1.752)	23.30 (1.112)	
Study population	62.7 (8.579)	24.20 (3.729)	25.82 (3.039)	

The average intraoperative blood loss for the study population was found to be 187.5 ± 42.888 mL. For those who underwent the procedure using electrocautery, it was found to be 222.67 ± 24.485 mL, while for those patients who underwent the procedure using the EBVS device, the average intraoperative blood loss was found to be 152.33 ± 24.166 mL. This result was found to be statistically significant (t = 11.198; p < 0.001; mean difference = 70.333 mL; dF = 58) (Table 2).

**Table 2:** Average intraoperative blood loss

Device	Mean intraoperative blood loss (mL)	SD
Electrocautery	222.67	24.485
EBVS device	152.33	24.166
Study population	187.5	42.888

The average duration of postoperative hospital stay for the study population was found to be 2.83 ± 1.564 days. For those who underwent the procedure using electrocautery, the average duration of postoperative hospital stay was found to be 2.93 ± 1.837 days, whereas for those patients who underwent the procedure using the EBVS device, the average postoperative hospital stay was found to be 2.73 ± 1.258 days. This was found to be statistically insignificant (t = 0.492; p = 0.625).

The volume of drainage in the postoperative period for the study population was found to be 625.67 ± 93.905 mL. For those who underwent the procedure using electrocautery, the average volume of drainage was found to be 659.33 ± 86.181 mL, whereas for those patients who underwent the procedure using the EBVS device, the average volume of drainage was found to be 632.00 ± 90.379 mL. This was found to be statistically insignificant. The duration of drainage in the postoperative period for the study population was found to be 11.38 ± 2.108 days.

**Figure 2:**
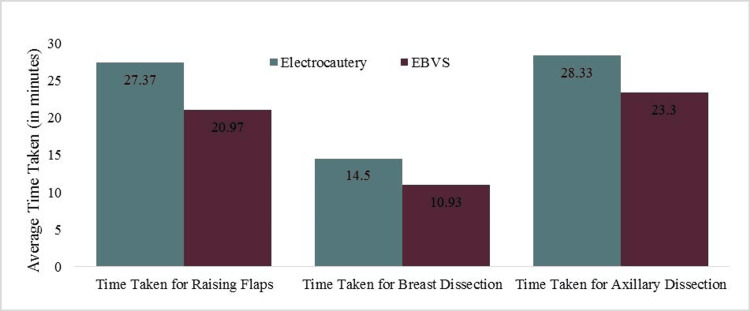
Comparison of surgical complications by device EVBS: electrothermal bipolar vessel sealing

The overall incidence of surgical complications in the study population was only 10%. There were four incidences (13.33%) of surgical complications in the group operated using electrocautery and only two incidences (6.67%) of surgical complications in the group operated using the EBVS device. This was not found to be statistically significant (c2 = 0.741; p = 0.389) (Figure 2).

The commonest complication was wound infection, with two incidences (6.67%) in the electrocautery group and one incidence (3.33%) in the EBVS group. There was no statistical difference in terms of the incidence of wound infections in either group (c2 = 0.351; p = 0.554).

There was a single incidence (3.33%) of seroma formation in the electrocautery group which was not observed in the EBVS device group. This was not found to be statistically significant (c2 = 1.017; p = 0.313).

The incidence of flap necrosis was equal in both groups with a single incidence (3.33%) in each group. Nerve injuries and hematoma formation were not observed amongst any of the study subjects in either group.

## Discussion

Surgical resection remains the keystone of breast cancer therapy, even though the specific approach for each case depends on the stage and type of tumor and includes a multidisciplinary tactic involving surgery, radiation, and chemotherapy. MRM is considered the most common operative procedure performed for advanced breast cancer [4]. Early complications in breast cancer surgery have always been attributed to excessive postoperative drainage, prolonged hospital stay, seroma formation, blood loss, hematoma formation, skin necrosis, and nerve injury [5]. In conventional practice, the elevation of skin flaps, removal of breast tissue from the pectoral wall, and axillary lymph node dissection have typically been conducted using either a scalpel for sharp dissection or electrocautery. Recently developed methods include the use of UltraCision dissection (harmonic scalpel) and tissue response generation using EBVS devices in raising these flaps.

The distribution of patients with node positivity in each device group was found to be comparable amongst the study subjects (c2 = 1.067; p = 0.302). This is in line with other similar studies that have emphasized keeping nodal positivity rates similar among both case and control groups, like the study by Chang et al. [6].

The key factor that determines the overall outcome is the comparison of total operative time, and devices with the ability to coagulate and cut play a significant role in reducing time and increasing the pace of surgery significantly [6].

This study revealed a difference of around 15 minutes in the total operative duration (p < 0.001), which is significant in terms of the time needed for general anesthesia and related complications. Correspondingly, statistically comparable timings were noticed in the various steps of MRM, which include the time taken for raising the flaps, the time taken for dissection of the breast tissue, and the time taken for dissection of the axilla [6].

The most rate-limiting step of this procedure is the axillary dissection due to the anatomical significance and nodal dissections. It was found that the use of these devices has a significant impact on the overall timings and the time taken for axillary dissection (p < 0.001 in each scenario). This is the key step in which these devices have an advantage over the other gadgets.

Few studies have analyzed the average intraoperative blood loss by various devices, and it was found of a significant reduction in total blood loss in the EBVS group (t = 11.198; p < 0.001) [7].

To evaluate the impact of energy devices on systemic inflammatory response, we measured the concentration of CRP on the third postoperative day. Our findings revealed that while CRP levels exhibited a typical increase in all patients, this elevation was not associated with the specific device utilized. This indicates that the type of electrosurgical device used for tissue dissection does not influence the systemic inflammatory response [8,9].

The rates of overall postoperative surgical complications, which include wound infections, flap necrosis, seroma formation, hematoma formation, and nerve injuries, were not found to be significantly different between the two device groups (p = 0.389) (Table 3).

**Table 3:** Comparison of the surgical complications in various studies EBVS: electrothermal bipolar vessel sealing

Complications	Device	This study	Study by Chang et al. [6]
Wound infections/skin necrosis	Electrocautery	3	12
	EBVS device	2	4
	p-value	0.640	0.264
Seroma formation	Electrocautery	1	7
	EBVS device	0	3
	p-value	0.313	0.734
Overall complications	Electrocautery	4	22
	EBVS device	2	7
	p-value	0.389	0.051

Limitations of the study

This is studied as a cross-sectional observational study, and the sample size is less. A more structured approach with a randomized study will benefit to come out with a better analysis of the outcome comparison.

## Conclusions

While the use of EBVS in MRM provides a considerable decrement in the total operative duration, duration of the various steps of surgery, intraoperative blood loss, and postoperative volume and duration of drainage, these devices have comparable outcomes in terms of postoperative complications or morbidity. These findings underscore the potential benefits of utilizing EBV to optimize surgical outcomes and improve patient care in MRM procedures.
